# The membrane cytoskeletal crosslinker ezrin is required for metastasis of breast carcinoma cells

**DOI:** 10.1186/bcr1006

**Published:** 2005-03-21

**Authors:** Bruce E Elliott, Jalna A Meens, Sandip K SenGupta, Daniel Louvard, Monique Arpin

**Affiliations:** 1 Division of Cancer Biology and Genetics, Cancer Research Institute, Queen's University, Kingston, Ontario, Canada; 2 Department of Pathology and Molecular Medicine, Queen's University, Kingston, Ontario, Canada; 3 Laboratory of Morphogenesis and Cell Signalling, UMR144 CNRS-Institut Curie, Paris, France

## Abstract

**Introduction:**

The membrane cytoskeletal crosslinker ezrin participates in several functions including cell adhesion, motility and cell survival, and there is increasing evidence that it regulates tumour progression. However, the role played by ezrin in breast cancer metastasis has not been clearly delineated.

**Methods:**

We examined the role of ezrin in metastasis using a highly metastatic murine mammary carcinoma cell line, namely AC2M2. Stable cell clones that overexpress wild-type ezrin or a dominant-negative amino-terminal domain of ezrin were selected. They were then tested for cell motility and invasion *in vitro*, and metastasis in a mouse *in vivo *tumour transplantation model.

**Results:**

Parental AC2M2 cells and cells overexpressing wild-type ezrin were transplanted into the mammary fat pad of syngeneic recipient mice; these animals subsequently developed lung metastases. In contrast, expression of the dominant-negative amino-terminal ezrin domain markedly inhibited lung metastasis. Consistent with this effect, we observed that the expression of amino-terminal ezrin caused strong membrane localization of cadherin, with increased cell–cell contact and a decrease in cell motility and invasion, whereas cells expressing wild-type ezrin exhibited strong cytoplasmic expression of cadherins and pseudopodia extensions. In addition, inhibitors of phosphatidylinositol 3-kinase and c-Src significantly blocked cell motility and invasion of AC2M2 cells expressing wild-type ezrin. We further found that overexpression of amino-terminal ezrin reduced levels of Akt pS473 and cytoskeletal-associated c-Src pY418 in AC2M2 cells, which contrasts with the high levels of phosphorylation of these proteins in cells expressing wild-type ezrin. Phosphorylated Erk1/2 was also reduced in amino-terminal ezrin expressing cells, although a mitogen-activated protein kinase kinase (MEK) inhibitor had no detectable effect on cell motility or invasion in this system.

**Conclusion:**

Our findings indicate that ezrin is required for breast cancer metastasis, and that c-Src and phosphatidylinositol 3-kinase/Akt are effectors of ezrin in the cell motility and invasion stages of the metastatic process. Together, these results suggest that blocking ezrin function may represent a novel and effective strategy for preventing breast cancer metastasis.

## Introduction

Deregulation of cell–cell contact, increased cell motility and invasion of carcinoma cells are key steps in the metastatic cascade [1], but the rate-limiting signalling steps that regulate this process in early-stage breast cancer have not yet been identified. One important molecule is the membrane cytoskeletal crosslinker protein ezrin, a member of the ezrin–radixin–moesin family, which is well documented to regulate several cytoskeletal-related functions, including cell adhesion, cell survival and cell motility [2-6]. There is also increasing evidence that ezrin regulates tumour progression [3]. Comparison of gene expression profiles in paired metastatic and nonmetastatic tumour cell lines and tissue samples revealed a strong increase in ezrin expression in metastases from rodent mammary and human pancreatic and colorectal carcinomas [7,8]. Likewise, ezrin exhibited strong expression in a variety of invasive human cancers, including osteosarcomas, melanomas, astrocytic tumours, and pancreatic, lung and endometrial carcinomas [9-12]. Further studies have indicated that suppression of ezrin protein function abrogates pulmonary metastases of murine rhabdomyosarcoma [13] and osteosarcoma cells [14], suggesting that ezrin may be a key regulatory molecule in malignant disease. However, the role played by ezrin in breast cancer metastasis has not been delineated.

Ezrin is regulated by an intramolecular association of its amino-terminal and carboxyl-terminal domains that masks their protein–protein binding sites [2]. Unfolding of the molecule into an active conformation occurs following binding to phosphoinositides and phosphorylation on the carboxyl-terminal threonine 567 [15]. The open molecule binds various membrane-associated adhesion molecules and ion exchangers to the amino-terminal region [2], and polymerized F-actin via the carboxyl-terminal domain [16]. Ezrin is involved in signal transduction pathways that depend on tyrosine kinases. Stimulation of cells with epidermal growth factor [17] or hepatocyte growth factor (HGF) [6] results in phosphorylation of ezrin primarily at two tyrosine residues (Tyr145 and Tyr353), which are important in regulating ezrin function. Phosphorylation of ezrin at these two tyrosine residues is required for tubulogenesis and motility [6], and Tyr353 regulates a phosphatidylinositol 3-kinase (PI3K)/Akt-dependent cell survival pathway through association with the p85 subunit of PI3K [5].

Our laboratory developed a mouse mammary carcinoma cell line, SP1, for studies of tumour progression and metastasis [18]. The parent SP1 cells form cadherin-based cell–cell contacts, exhibit oestrogen-dependent primary tumour growth following transplantation *in vivo*, and are poorly metastatic. Recently, we showed that ezrin acts cooperatively with activated c-Src in deregulating cadherin-based cell–cell contacts and scattering of SP1 cells [19]. We further showed that blocking ezrin function by overexpressing a truncated domain (amino-terminal amino acids 1–309) of ezrin, which has dominant-negative function [6], abrogates cell scattering and enhances cadherin-based cell–cell contacts in SP1 cells [19]. These findings prompted us to examine the role played by ezrin in cell invasion and metastasis of breast carcinoma cells. For this study, we used a highly metastatic variant cell line, namely AC2M2, selected from rare metastatic nodules of SP1 cells *in vivo *[18]. AC2M2 cells exhibit strong cytoplasmic localization of cadherins and extensive filopodia with weak cell–cell contacts. Our findings show that overexpression of the dominant negative amino-terminal ezrin mutant in AC2M2 cells abrogates *in vivo *metastasis and inhibits cell motility and invasion *in vitro*. Furthermore, cells overexpressing the amino-terminal ezrin mutant showed marked reduction in PI3K/Akt, Erk1/2 and c-Src activities, indicating a possible role for these signalling molecules as downstream effectors of ezrin in the metastatic process.

## Materials and methods

### Antibodies and reagents

Rabbit anti-sheep IgG conjugated with horseradish peroxidase was from Jackson ImmunoResearch Laboratories (West Grove, PA, USA). Mouse (monoclonal) anti-pan cadherin antibody was obtained from Sigma Immunochemicals (Oakville, Ontario, Canada). Alexa-488-conjugated goat anti-mouse IgG was obtained from ICN Biomedicals (Mississauga, Ontario, Canada). Mouse monoclonal antibody against the vesicular stomatitis virus glycoprotein (VSVG; clone P5D4) was obtained from Roche Diagnostics (Mississauga, Ontario, Canada). Rabbit anti-ezrin IgG (carboxyl-terminus specific) was prepared as described previously [6]. Antibodies against the phosphorylated forms of Akt pS473, Erk1/2 pT185/pY187 and c-Src pY418 (i.e. phospho-specific antibodies), and corresponding pan-Akt and pan-Erk1/2 antibodies were obtained from Medicorp (Montreal, Quebec, Canada). Pan c-Src antibody (Ab-1) was obtained from Oncogene Science (Cambridge, MA, USA), and Matrigel was obtained from Becton Dickinson Co. (Mississauga, Ontario, USA). The PI3K inhibitor LY294002, the c-Src inhibitor SU6656 and the mitogen-activated protein kinase (MAPK) kinase (MEK) inhibitor PD098059 were obtained from Calbiochem (San Diego, CA, USA).

### Cell lines and tissue culture

The SP1 tumour cell line was derived from a spontaneous, poorly metastatic murine mammary intraductal adenocarcinoma, isolated from a female CBA/J retired breeder [18]. AC2M2 cells are a highly metastatic variant selected from the SP1 cell line following three times serial passage of a lung metastatic nodule into the mammary fat pad of syngeneic mice, as described previously [18]. Cell lines were cultured in Dulbecco's modified Eagle medium (Invitrogen, Burlington, Ontario, Canada) supplemented with 7% foetal bovine serum.

### Cell transfection

The pCB6 vector containing cDNA encoding VSVG-tagged ezrin or the VSVG-tagged amino-terminal truncated domain (amino acids 1–309) of ezrin was previously described [6]. All transfections were carried out with Lipofectamine Plus reagent (Canadian Life Technology, Burlington, Ontario, Canada) in accordance with the manufacturer's instructions. Stable transfectants were selected with G418 (450 μg/ml; Sigma-Aldrich, Oakville, Ontario, Canada) and individual clones were isolated. Exogenous protein expression in each clone was confirmed using indirect immunofluorescence (data not shown) and semiquantitative western blot analysis.

### Indirect immunofluorescence

Indirect immunofluorescence staining was conducted as previously described [19]. Briefly, cells were plated overnight on cover slips, fixed in 3% paraformaldehyde/phosphate-buffered saline (PBS), permeabilized with 0.2% Triton X-100 and blocked for 30 min with 3% bovine serum albumin. Cells were incubated with anti-cadherin antibody, followed by the appropriate secondary antibody. Preparations were observed using a Leica TCS SP2 confocal microscope (Leica Microsystems, Richmond Hill, Ontario, Canada) in the Queen's Cancer Research Institute and Protein Discovery and Function Facility. Image acquisitions were processed using Adobe Photoshop software.

### Western blotting

Cells were grown to 60% confluence in six-well tissue culture plates (NUNC, Mississauga, Ontario, Canada), rinsed with ice-cold PBS with 0.1 μmol/l CaCl_2 _and 0.1 μmol/l MgCl_2 _(PBS*), and lysed in 2× Laemmli buffer. For blotting with phospho-specific antibodies, cells were serum-starved overnight and plated on fibronectin-coated (10 mg/ml) plates for the times indicated. For analysis of c-Src, the cytoskeletal fraction was first extracted by a 1-min incubation with 250 μl of a Triton X-100 buffer (soluble fraction) that preserves cytosketal-associated material (csk buffer: 50 mmol/l MES, 3 mmol/l EGTA, 5 mmol/l MgCl_2_, 0.5% Triton X-100; pH 6.4). The remaining cellular material (insoluble fraction) was rinsed quickly with 500 μl csk buffer, and was further extracted with 250 μl 2× Laemmli buffer. Protein determination of cell lysates was performed using a DC protein assay kit (Biorad, Mississauga, Ontario, Canada). All cell lysates were subjected to 10% SDS-PAGE under reducing conditions (with 2.5% β_2_-mercaptoethanol) and transferred to PVP (polyvinylpyrrolidone) membranes. The membranes were blocked and probed with the appropriate primary and secondary antibodies, followed by chemiluminescence with the Northern Lightning™ reagent (Perkin Elmer Life Sciences Inc., Boston, MA, USA). Semiquantitation of exogenous versus endogenous ezrin expression was determined by western blotting of serial dilutions of total cell lysates (0.6–20 μg), as described previously [6,19]. The fold increase in exogenous ezrin expression was determined by comparing the titration end-point of the corresponding ezrin band in each clone with that of cells transfected with empty pCB6 vector. For amino-terminal ezrin expressing clones, the ratio of VSVG–amino terminal ezrin to endogenous ezrin normalized to actin was calculated using densitometric analysis.

### Tumour transplantation and metastasis

SP1 and AC2M2 cell lines were injected (7.5 × 10^3 ^cells in 10 μl/mouse) into the mammary fat pad of syngeneic mice, as described previously [18]. Primary tumour growth was monitored every 2–3 days, and metastasis in lung, viscera, liver and draining lymph nodes was assessed 6 weeks later. Histological analysis of tissue sections stained with haematoxylin and eosin was performed to confirm the presence of metastases in the various organs. Based on the gross and histological analyses, animals were assessed as positive or negative with respect to metastasis. At least eight mice were included in each group.

### Wound healing assay

Cells were plated onto 12-well tissue culture dishes (NUNC) at near confluence in complete tissue culture medium. Confluent cells were scored using a 20 μl Eppendorf micropipette tip. The medium was immediately replaced, and spontaneous cell migration was monitored using a Nikon inverted microscope for 18–24 hours, as indicated. For experiments comparing transfected clones, analysis at 18 hours is shown because maximal differences between groups were observed at this time. For experiments with pharmacological inhibitors, the assay was allowed to proceed for 24 hours to optimize the effect of the inhibitors. Viability of cells was maintained during this assay period. Phase contrast images were captured, and the distance of wound closure (compared with control at t = 0 hours) was measured in three independent wound sites per group. Relative cell motility was calculated as the wound width at t = 0 hours minus the wound width at t = 18–24 hours, as indicated. Values from at least three independent experiments were pooled and expressed as mean ± standard deviation (SD).

### Invasion assay

Transfected AC2M2 cells were plated in 24-well transwell cultures (NUNC). Cells (5 × 10^4^) were overlayered in 200 μl of 0.5% foetal bovine serum/Dulbecco's modified Eagle medium on Matrigel-coated transwell membranes (8 μm pore size), and with 0.5 ml of complete medium in the lower chamber. After 36–48 hours (as indicated) the cells were fixed and stained with Harris's modified haematoxylin (Fisher Scientific, Nepean, Ontario, Canada), and noninvading cells on the top of the membrane were removed using a Q-tip. The membranes were then mounted on glass slides, and images corresponding to the entire membrane surface were captured using an Olympus inverted microscope equipped with a CCD camera (Apogee Instruments Inc., Auburn, CA, USA). The total numbers of cells invading through the membrane were quantitated using ImagePro software (Symbol Technologies, Mississauga, Ontario, Canada). Values were normalized to empty pCB6 vector group in each experiment, and the results from at least three independent experiments were pooled and expressed as mean relative cell invasion ± SD.

### Statistical analysis

Statistical significance among metastasis groups was determined using the two-sided Fisher's exact test. The day at which tumours reached 1 cm diameter was determined by linear regression analysis of growth curves from individual mice, and expressed as mean ± SD. Statistical significance between groups in the motility and invasion assays was assessed using a Fisher's two-tailed t-test with Microsoft Excel software.

## Results

### Overexpression of amino-terminal ezrin inhibits metastasis of AC2M2 breast carcinoma cells

**Figure 1:**
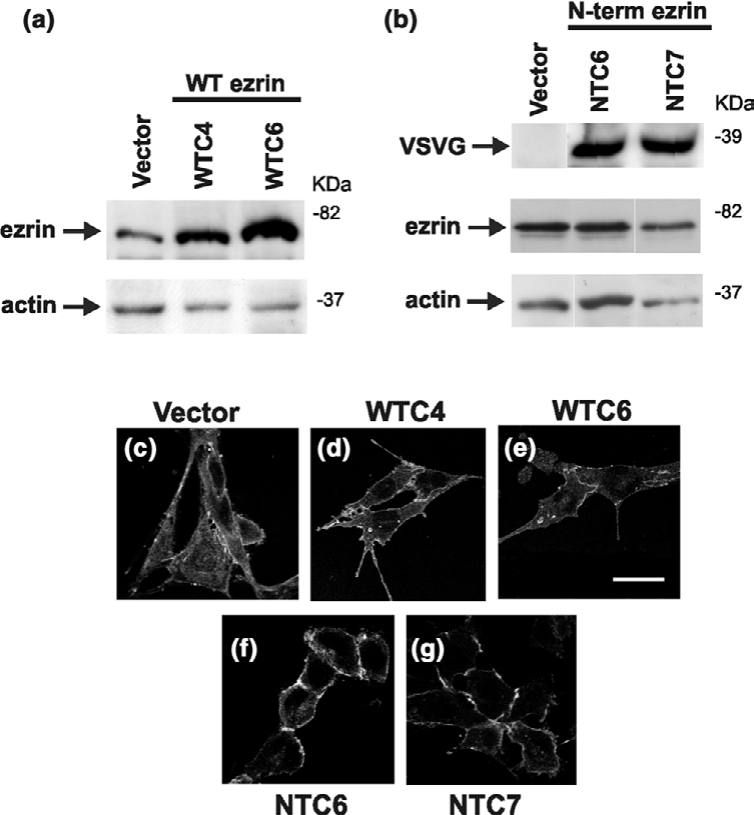
Localization of cadherins in metastatic mammary carcinoma cells overexpressing wild-type and amino-terminal ezrin. Metastatic AC2M2 cells were transfected with empty pCB6 vector, or a vector encoding wild-type or amino-terminal ezrin, as described in the text. **(a) **Serial dilutions of the total cell extracts (0.6–20 μg) were subjected to reduced 10% SDS-PAGE and transferred to PVP membranes. The membranes were probed with anti-ezrin and anti-actin antibodies, followed by the appropriate peroxidase-conjugated secondary antibodies, and developed with chemiluminescence. Lanes from left to right contained 10 μg of the following cell extracts: pooled AC2M2 cells transfected with empty pCB6 vector, and two clones transfected with wild-type (WT) ezrin. WTC4 and WTC6 exhibited 4-fold and 8-fold overexpression, respectively, of ezrin compared with vector control cells. **(b) **Membranes were probed with anti-vesicular stomatitis virus glycoprotein (VSVG), anti-ezrin and anti-actin antibodies. Lanes contained 15 μg of cell extracts from pooled pCB6-transfected cells, and two clones transfected with amino-terminal ezrin. Clones NTC6 and NTC7 exhibited 1.6-fold and 4.6-fold amino-terminal ezrin expression, respectively, compared with endogenous ezrin, as determined by densitometric analysis of VSVG and ezrin blots normalized to actin. **(c-g) **The above cell lines were immunostained with anti-pan cadherin antibody, as described the text. Representative confocal microscope images are shown.

We previously showed that overexpression of a truncated amino-terminal domain of ezrin blocks HGF-induced migration and morphogenesis of epithelial cells [6], and reduces cell scattering in SP1 carcinoma cells expressing activated c-Src [19]. We therefore examined the effect of amino-terminal ezrin on invasion and dissemination of a highly metastatic mammary carcinoma variant cell line, namely AC2M2, which is derived from SP1 cells. We generated stable transfectants of AC2M2 cells expressing VSVG-tagged wild-type and amino-terminal ezrin in a pCB6 eukaryotic expression vector, as described previously [6]. Ezrin protein levels in clones transfected with pCB6 vector containing wild-type ezrin were found to be increased approximately 4-fold and 8-fold, respectively, in WTC4 and WTC6 cells compared with cells transfected with empty vector, as determined by semiquantitative western blotting (Fig. 1a). Expression of amino-terminal ezrin was increased 1.6-fold and 4.5 fold, respectively, in NTC6 and NTC7 cells compared with endogenous ezrin and normalized to actin, as determined by densitometric analysis (Fig. 1b). AC2M2 cells transfected with empty pCB6 vector, or overexpressing wild-type ezrin exhibited strong cytoplasmic expression of cadherins and filopodia extensions (Fig. 1c–e). In contrast, overexpression of amino-terminal ezrin expression caused strong membrane localization of cadherins with increased cell–cell contacts (Fig. 1f,g).

To assess the role of ezrin function in metastasis, clones of AC2M2 cells overexpressing wild-type ezrin or amino-terminal ezrin were injected into the mammary fat pad of syngeneic female mice, and metastases were assessed 6 weeks after injection (Table 1). No change in primary tumour growth rate was observed, as assessed by percentage primary tumour take and day of 1 cm tumour diameter, except for one amino-terminal expressing clone (NTC6), which showed reduced tumour growth rate. To compensate, mice in this group were killed approximately 1 week later to allow all tumours to grow to an equivalent size. Untransfected AC2M2 cells exhibited extensive pulmonary metastases (10/11), as compared with the poorly metastatic parent SP1 cells (3/13; *P *= 0.003). Pooled AC2M2 cells transfected with empty pCB6 vector (7/8) or two clones overexpressing wild-type ezrin (13/15 and 6/7) were also strongly metastatic. In contrast, expression of amino-terminal ezrin caused a marked reduction in metastases in two independent clones (0/8 and 3/8; *P *< 0.0001 and *P *= 0.002, respectively). Similar results were obtained with an additional amino-terminal ezrin overexpressing clone (NTB8) from an independent transfection (0/5; *P *= 0.002). Analysis of pooled results showed that metastases in the three amino-terminal ezrin groups (3/21) were strongly reduced compared with the two wild-type ezrin groups (19/22; *P *< 0.0001).

**Figure 2:**
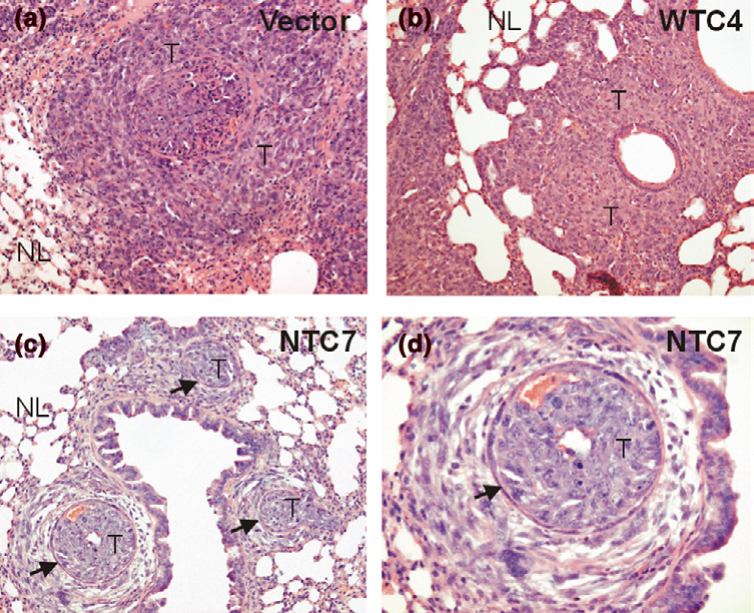
Expression of amino-terminal ezrin significantly reduces lung metastases of carcinoma cells. Metastatic AC2M2 cells transfected with empty pCB6 vector or a vector encoding wild-type ezrin or amino-terminal ezrin were injected (7.5 × 10^3^/mouse) into the mammary fat pad of syngeneic mice, and metastasis was assessed 6 weeks later, as described in the text. Images are shown from tissue sections from the lungs of mice injected with AC2M2 cells transfected with **(a) **empty pCB6 vector, **(b) **wild-type ezrin (WTC4), or **(c,d) **amino-terminal ezrin, stained with haematoxylin and eosin. Normal lung tissue is indicated by 'NL'. Tumour metastases are indicated by 'T'. Arrows indicate endothelial lining of vascular channels with tumour emboli. Original magnifications: panels a–c, 200×; panel d, 400×.

Histological analysis of various organ sites in animals with tumours transfected with empty pCB6 vector or wild-type ezrin (WTC4, WTC6) revealed massive tumour nodules in the lung (Fig. 2a,b), as well as occasional metastases in the small intestine (data not shown). In contrast, the majority of mice injected with tumour cells overexpressing amino-terminal ezrin (NTC6, NTC7, NTB8) showed no metastatic lesions; the few metastases that did form (in the NTC7 group) were generally smaller and primarily localized to vascular channels (Fig. 2c,d). These findings suggest that ezrin function is necessary for metastasis in this breast cancer model.

### Overexpression of amino-terminal ezrin inhibits cell motility and invasion of AC2M2 cells

**Figure 3:**
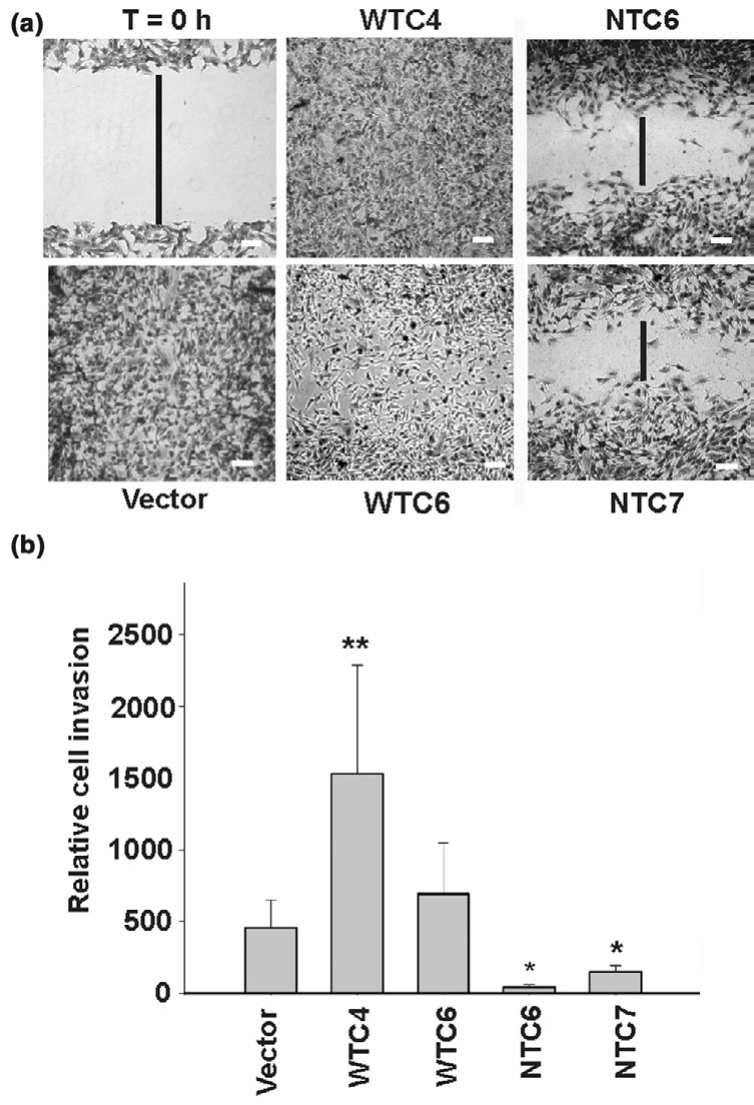
Expression of amino-terminal ezrin inhibits motility and invasion of carcinoma cells. **(a) **Transfected AC2M2 clones (see Fig. 1) were grown to confluence in 12-well NUNC tissue culture plates with 2 ml of 10% foetal bovine serum (FBS)/Dulbecco's modified Eagle medium (DMEM). Cultures were wounded by streaking cell monolayers with a 20 μl Eppendorf micropipette tip and monitored up to 24 hours, as described in the text. Representative fields photographed after 18 hours are shown. Vertical bars indicate the wound distance. Bar, 50 μm. **(b) **Transfected AC2M2 cells were subjected to an invasion assay, as described in the text. Cells (5 × 10^4^) were over-layered in 200 μl of 0.5% FBS/DMEM medium on the transwell membranes (8 μm pore size), with 0.5 ml of complete medium in the lower chamber. After 36–48 hours, cells were fixed and stained with modified haematoxylin, and cells invading through the membrane were counted using ImagePro software, as described in the text. The numbers of invading cells were normalized to empty pCB6 vector group in each experiment. The results from at least three independent experiments were pooled, and expressed as the mean relative cell invasion ± standard deviation. Asterisks indicate (**) a significant increase or (*) a significant reduction in cell invasion compared with vector control, using a two-sided Fisher's t-test. *P *= 0.03 for WTC4**; *P *= 0.009 for NTC6*; and *P *= 0.02 for NTC7*.

Metastasis is a multistep process involving intravasation, transport through the vasculature or lymphatics, and extravasation into target organs [20]. Previous studies indicated a role for ezrin in HGF-induced cell scattering and migration [6,19]. We therefore examined the role played by ezrin in cell motility and invasion of metastatic AC2M2 cells. Wound healing assays were conducted using AC2M2 cells transfected with empty pCB6 vector or a vector encoding wild-type ezrin or amino-terminal ezrin. A wound was scored on a cell monolayer, and the wound closure was assessed at various times up to 24 hours. Our results show that expression of amino-terminal ezrin reduced the ability of AC2M2 cells to close the wound by approximately 2.5-fold compared with cells transfected with empty pCB6 vector or with wild-type ezrin (Fig. 3a). Invasion assays were carried out using Matrigel-coated transwell culture chambers, and invading cells were counted after 36–48 hours using image analysis. AC2M2 cells expressing wild-type ezrin showed increased cell invasion compared with cells transfected with empty pCB6 vector, whereas amino-terminal ezrin expressing clones exhibited markedly reduced cell invasion (Fig. 3b).

### PI3K and c-Src are required for ezrin-mediated cell motility and invasion of AC2M2 cells

**Figure 4:**
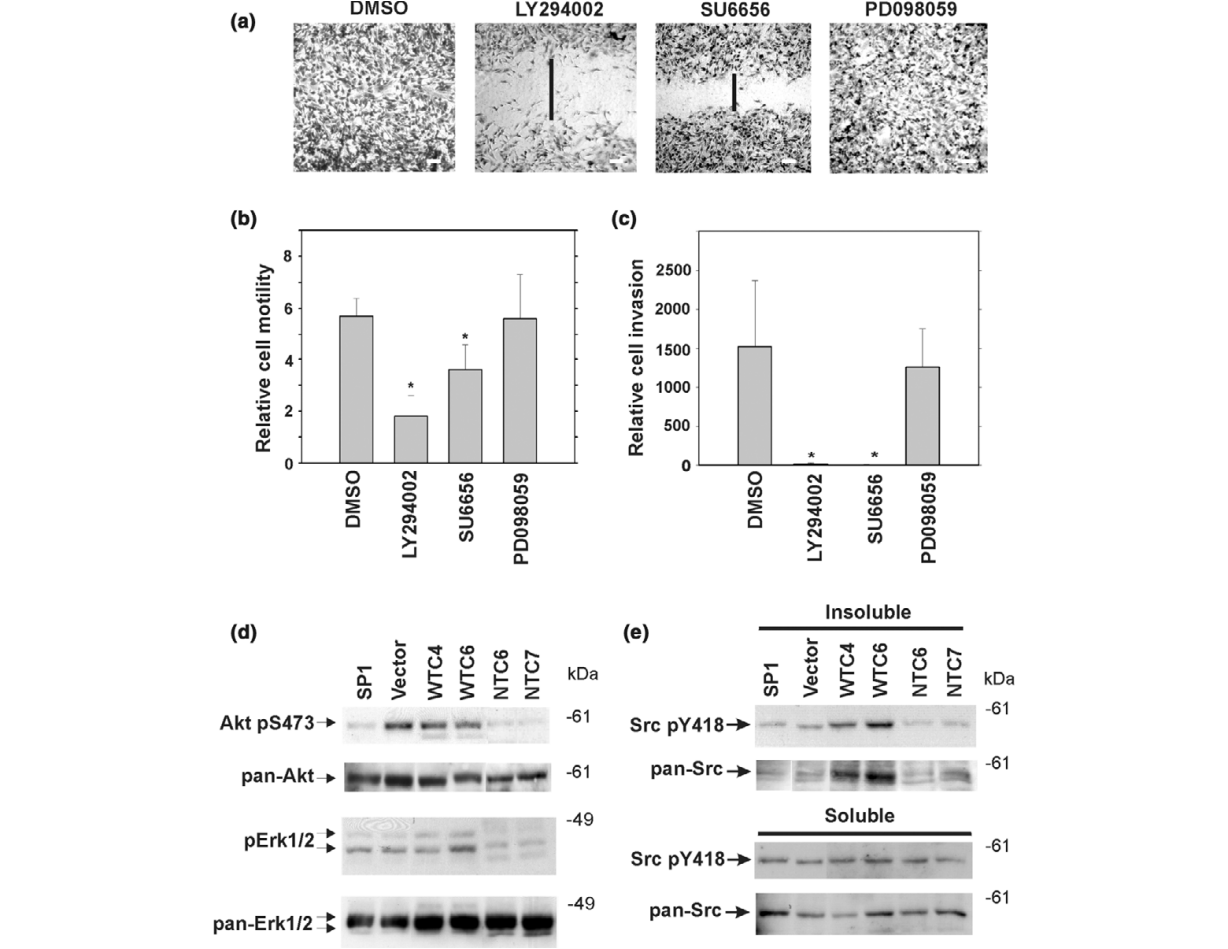
Role of phosphatidylinositol 3-kinase (PI3K) and c-Src in ezrin-mediated cell motility and invasion. **(a) **In a wound healing experiment with WTC4 cells, the PI3K inhibitor LY294002 (10 μmol/l), the c-Src inhibitor SU6656 (10 μmol/l), or the mitogen-activated protein kinase kinase (MEK) inhibitor (PD098059; 30 μmol/l), or the solvent DMSO (dimethyl sulfoxide; 10 μl/culture) was added to cultures, and wound closure was monitored up to 24 hours, as described for Fig. 3a. Representative fields photographed after 24 hours are shown. **(b) **The histogram shows pooled results from clones WTC4 and WTC6 in three independent wound healing experiments with the above inhibitors. Significant reduction in motility was observed in groups treated with LY294002 (*P*= 0.002) and SU6656 (*P *= 0.03). **(c) **WTC4 cells were set up in transwell cultures with PI3K, c-Src, or MEK inhibitors at the concentrations indicated above, and cell invasion was assessed after 36 hours. Results are expressed as the mean cell invasion ± standard deviation of at least three independent experiments. Single asterisk (*) indicates a specific reduction in invasion compared with DMSO-treated cells (LY294002, *P *= 0.02; SU6656, *P *= 0.02). **(d) **For analysis of Akt and Erk1/2 activation, transfected AC2M2 cell lines (see Fig. 1a) were serum starved overnight and cultured on fibronectin substratum (10 μg/ml) for 45 min. Cells were then lysed, and equal protein amounts of each cell lysate were subjected to 10% SDS-PAGE under reduced conditions. Proteins were transferred to PVP membranes, and western blotting was carried out with antibodies against Akt pS473, pan-Akt, Erk1/2 pT185/pY187 and pan-Erk1/2. **(e) **For c-Src analysis, cells were plated for 2 hours on fibronectin substratum and cell lysates of Triton X-100 soluble and insoluble (cytoskeletal-associated) fractions were prepared, as described in the text. Blots were probed with antibodies against c-Src pY418 and pan c-Src.

PI3K, c-Src and MAPK pathways have been implicated in cell motility and invasion in many cell types [21]. As a first step in unravelling the signalling pathways involved in cell motility and invasion of AC2M2 cells overexpressing wild-type ezrin, we determined the effect of specific signal transduction inhibitors on these functions. The results showed that the PI3K inhibitor LY294002 markedly attenuated cell motility (3-fold) of two clones overexpressing wild-type ezrin as well as cells transfected with empty pCB6 vector (data not shown; Fig. 4a,b). The c-Src inhibitor SU6656 had a moderate (1.5-fold) blocking effect on cell motility. In contrast, the MEK inhibitor PD098059 had no detectable effect. In addition, cell invasion was dramatically inhibited by both PI3K and c-Src inhibitors, but not by the MEK inhibitor (Fig. 4c). All three inhibitors at the concentrations indicated were previously shown to block activity of the respective kinases, as determined by western blotting with the corresponding phospho-specific antibodies [22] (data not shown). Thus, PI3K and c-Src pathways, but not the MAPK pathway, are required for both cell motility and invasion of wild-type ezrin-expressing AC2M2 cells.

### Overexpression of amino-terminal ezrin abrogates signalling through PI3K/Akt, c-Src, and MAPK pathways in AC2M2 cells

The results shown in Fig. 4a–c raise the possibility that PI3K and c-Src are downstream of ezrin in the regulation of cell motility and invasion in AC2M2 cells. To investigate this notion, we examined the effect of amino-terminal ezrin on phosphorylation of Akt S473 (a downstream effector of PI3K [5]), c-Src Y418 (within the c-Src catalytic domain [1]) and Erk1/2 T185/Y187 (within the activation loop of Erk1/2; Fig. 4d). Serum-starved AC2M2 cells were plated on fibronectin for the times indicated, lysed, and subjected to western blotting with the appropriate phospho-specific antibodies. Interestingly, expression of Akt pS473 was increased in metastatic AC2M2 cells (empty vector) compared with the poorly metastatic parental SP1 cells. Overexpression of amino-terminal ezrin, compared with wild-type ezrin, markedly reduced the level of Akt pS473 in AC2M2 cells, indicating regulation by ezrin of the PI3K/Akt pathway in these cells. In parallel, the level of phospho-Erk1/2 (pT185/pY187) was sustained in AC2M2 cells transfected with empty pCB6 vector or wild-type ezrin, and was reduced in cells expressing amino-terminal ezrin. Because activated c-Src associates with its substrate at the focal adhesion complex [1,23], we examined c-Src pY418 in both the Triton X-100 soluble and insoluble (cytoskeletal-associated) fractions in AC2M2 cells (Fig. 4e). Our results show that the levels of cytoskeletal-associated total c-Src and c-Src pY418 were increased in cells overexpressing wild-type ezrin, but were markedly reduced in cells expressing amino-terminal ezrin. In contrast, the level of c-Src pY418 in the soluble fraction (and in total cell lysates; data not shown) remained unchanged in all cell groups. Thus, ezrin plays a key role in stabilizing the activities of PI3K and c-Src, as well as Erk1/2.

## Discussion

In the present study we demonstrate for the first time that ezrin function is required for metastasis of breast carcinoma cells. Our results show that inactivating ezrin function by overexpressing a dominant-negative (amino-terminal) ezrin mutant blocks spontaneous pulmonary metastases of mammary carcinoma cells transplanted into the orthotopic site. We further show that overexpression of wild-type ezrin increases carcinoma cell invasion, whereas amino-terminal ezrin causes reduced cell scattering, motility and invasion, thus indicating a possible mechanism by which ezrin regulates progression to invasive cancer. Similar reports have shown that overexpressing ezrin antisense [13] or an ezrin T567A dominant-negative mutant [14] blocks both experimental and spontaneous metastasis of murine rhabdomyosarcoma and osteosarcoma cells, and in the latter report the rate-limiting effect was demonstrated to be on early survival of metastastic cells. Thus, ezrin may have multiple effects on the metastatic cascade.

Moreover, we found that overexpression of wild-type ezrin does not augment metastasis of parental SP1 cells. Furthermore, no increase in expression of endogenous ezrin was observed in the metastatic (AC2M2) compared with the parent SP1 cell lines (data not shown). These findings imply that overexpression of ezrin alone is not sufficient to induce metastasis in this tumour model, suggesting that multiple pathways are involved in the metastatic cascade. However, it is difficult to relate quantitative changes in exogenous ezrin overexpression directly with dominant active or negative functional effects, because the signalling networks involved are complex and the functional assays are long term (18–36 hours for cell motility and invasion, and 5 weeks for metastasis). Our focus was therefore on the qualitative effects on breast cancer metastasis of blocking ezrin function using a dominant-negative amino-terminal ezrin mutant [6].

Our finding that Akt S473 phosphorylation is enhanced in AC2M2 cells compared with parental SP1 cells suggests a key role for the PI3K/Akt pathway in metastasis. Expression of amino-terminal ezrin reduced the levels of Akt S473 phosphorylation to that of SP1 cells, indicating a dominant regulatory effect of ezrin on PI3K/Akt signalling in AC2M2 cells. Furthermore, inhibition of PI3K blocked both cell motility and invasion in AC2M2 cells overexpressing wild-type ezrin, indicating that PI3K is a downstream effector of ezrin in these functions. In addition to its role in cell motility via PI3K, ezrin may also participate in metastasis by increasing cell survival. Indeed, we previously showed that ezrin signals cell survival by activating the PI3K/Akt pathway [5].

Interestingly, although Erk1/2 activation is also reduced in amino-terminal ezrin-expressing cells, inhibition of the MAPK pathway has no detectable effect on cell motility or invasion in this tumour model. However, previous reports have indicated that an activated MEK mutant can rescue early survival of metatastic osteosarcoma cells expressing ezrin antisense [14]. It is therefore possible that an ezrin-dependent MAPK pathway still plays a role in our breast metastasis model, as demonstrated by Khanna and coworkers [14].

We also observed a strong increase in cytoskeletal-associated c-Src pY418 in cells overexpressing wild-type ezrin, and this effect was abrogated in cells expressing amino-terminal ezrin. Furthermore, inhibition of c-Src activity partially blocks cell motility and completely abrogates invasion of ezrin-expressing AC2M2 cells. These findings are consistent with our previous demonstration [24] of a reciprocal relationship between c-Src and ezrin in phosphorylation/activation of these two proteins, and their role in regulating cell spreading and cell migration. The interactive role played by ezrin with c-Src in cell adhesion-dependent functions may provide an important mechanism by which integrin signals are amplified through the cytoskeleton. Previous findings from our laboratory [25] and others [26] have shown that inhibition of specific integrin function can block metastasis of breast carcinomas. The findings presented here raise the possibility that ezrin and c-Src are key regulators of integrin-dependent steps in cell invasion and metastasis.

Because both PI3K and c-Src are key effectors downstream of ezrin in the cell motility and invasive phenotypes, these signalling pathways are likely to be rate limiting in the regulation by ezrin of metastatic progression *in vivo*. In addition, cooperativity between PI3K and c-Src may be important in regulating ezrin function in cell motility and invasion, for example through activation of Rho GTPases [4,13,21]. In addition, interaction of ezrin with other signalling molecules such as the Na^+^/H^+ ^exchanger regulatory factor (NHERF-1), recently described to be altered in breast cancer, may also be involved [27,28]. Further investigation is required to assess the relevance of these downstream pathways in breast metastasis.

## Conclusion

In the present study we show for the first time that ezrin is required for invasion and metastasis of mammary carcinoma cells. We further show that PI3K and c-Src activities are modulated by ezrin and are required for ezrin-dependent cell invasion. Because we recently showed that c-Src is also upstream of ezrin [24], and acts cooperatively with ezrin in deregulating cell–cell contacts and cell scattering [19], we propose that coordinate upregulation of ezrin and c-Src activity may be a key regulatory step in metastatic breast disease. Together, our findings suggest that ezrin activation may represent an effective prognostic marker and a potential target for treatment of invasion and metastasis of human breast cancer.

## Abbreviations

HGF = hepatocyte growth factor; MAPK = mitogen-activated protein kinase; MEK = mitogen-activated protein kinase kinase; PBS = phosphate-buffered saline; PI3K = phosphatidylinositol 3-kinase; SD = standard deviation; VSVG = vesicular stomatitis virus glycoprotein.

## Competing interests

The author(s) declare that they have no competing interests.

## Authors' contributions

BEE carried out the tumour transplantation and metastasis studies and the cell motility, and wrote the manuscript. JAM performed invasion assays and western blotting studies, and assisted with the tumour transplantation studies. SKS performed the pathology on tissue sections. MA and DL participated in the design of the study, provided the wild-type and amino-terminal ezrin pCB6 expression vectors, and assisted in writing and editing the manuscript. All authors read and approved the final manuscript.

## Figures and Tables

**Table 1:** Expression of amino-terminal ezrin inhibits metastasis of breast carcinoma cells

Cell line^a^	Transfected with	Primary tumour takes (%)	Day of 1 cm tumour diameter^b^	Day of sacrifice	Metastasis (%)^c^
SP1	None	100% (13/13)	24 ± 7	35	23% (3/13)
AC2M2	None	100% (11/11)	30 ± 5	41	90% (10/11)^†^
					
pCB6	Vector	100% (8/8)	33 ± 4	39	88% (7/8)
					
WTC4	WT ezrin	100% (7/7)	28 ± 4	41	88% (6/7)
WTC6	WT ezrin	100% (15/15)	26 ± 2	39	87% (13/15)
					
NTC6	N-term ezrin	38% (3/8)	40 ± 10*	47	0% (0/8)^‡^
NTC7	N-term ezrin	100% (8/8)	28 ± 1	39	38% (3/8)^‡^
NTB8^d^	N-term ezrin	100% (5/5)	29 ± 3	41	0% (0/5)^‡^

^a^Poorly metastatic parental SP1 cells or highly metastatic variant AC2M2 cells alone, or transfected with empty pCB6 vector, or a vector encoding wild-type (WT) ezrin or amino-terminal (N-term) ezrin, were transplanted (7.5 × 10^3 ^cells) into the mammary fat pad of syngeneic mice (see text). ^b^Day to 1 cm tumour diameter was calculated by linear regression analysis of data from individual mice. Values are expressed as mean ± standard deviation. Clone NTC6 showed a significant increase (*) in the day of 1 cm tumour diameter compared with WTC4 and WTC6 (*P *= 0.012). Mice with NTC6 tumours were therefore killed approximately 1 week later to allow tumour growth to a comparable size. ^c^AC2M2 cells showed significantly more metastases than did the parental SP1 cells (^†^*P *= 0.003; Fisher's exact test). Pooled results from three N-term ezrin expressing clones showed a significant reduction in metastases compared with two WT ezrin expressing clones (^‡^*P *< 0.0001). Individual *P *values for NTC6, NTC7 and NTB8 are as follows (respectively): <0.0001, 0.002 and 0.002. ^d^NTB8 is an N-term ezrin-expressing clone derived from an independent transfection of AC2M2 cells, and was transplanted as described above.

## References

[B1] Frame MC (2004). Newest findings on the oldest oncogene; how activated Src does it. J Cell Sci.

[B2] Bretscher A, Edwards K, Fehon RG (2002). ERM proteins and merlin: integrators at the cell cortex. Nat Rev Mol Cell Biol.

[B3] Gautreau A, Louvard D, Arpin M (2002). ERM proteins and NF2 tumor suppressor: the Yin and Yang of cortical actin organization and cell growth signaling. Curr Opin Cell Biol.

[B4] Pujuguet P, Del Maestro L, Gautreau A, Louvard D, Arpin M (2003). Ezrin regulates E-cadherin-dependent adherens junction assembly through Rac1 activation. Mol Biol Cell.

[B5] Gautreau A, Poullet P, Louvard D, Arpin M (1999). Ezrin, a plasma membrane-microfilament linker, signals cell survival through the phosphatidylinositol 3-kinase/Akt pathway. Proc Natl Acad Sci USA.

[B6] Crepaldi T, Gautreau A, Comoglio PM, Louvard D, Arpin M (1997). Ezrin is an effector of hepatocyte growth factor-mediated migration and morphogenesis in epithelial cells. J Cell Biol.

[B7] Khanna C, Khan J, Nguyen P, Prehn J, Caylor J, Yeung C, Trepel J, Meltzer P, Helman L (2001). Metastasis-associated differences in gene expression in a murine model of osteosarcoma. Cancer Res.

[B8] Nestl A, Von Stein OD, Zatloukal K, Thies WG, Herrlich P, Hofmann M, Sleeman JP (2001). Gene expression patterns associated with the metastatic phenotype in rodent and human tumors. Cancer Res.

[B9] Geiger KD, Stoldt P, Schlote W, Derouiche A (2000). Ezrin immunoreactivity is associated with increasing malignancy of astrocytic tumors but is absent in oligodendrogliomas. Am J Pathol.

[B10] Tokunou M, Niki T, Saitoh Y, Imamura H, Sakamoto M, Hirohashi S (2000). Altered expression of the ERM proteins in lung adenocarcinoma. Lab Invest.

[B11] Ohtani K, Sakamoto H, Rutherford T, Chen Z, Satoh K, Naftolin F (1999). Ezrin, a membrane-cytoskeletal linking protein, is involved in the process of invasion of endometrial cancer cells. Cancer Lett.

[B12] Makitie T, Carpen O, Vaheri A, Kivela T (2001). Ezrin as a prognostic indicator and its relationship to tumor characteristics in uveal malignant melanoma. Invest Ophthalmol Vis Sci.

[B13] Yu Y, Khan J, Khanna C, Helman L, Meltzer PS, Merlino G (2004). Expression profiling identifies the cytoskeletal organizer ezrin and the developmental homeoprotein Six-1 as key metastatic regulators. Nat Med.

[B14] Khanna C, Wan X, Bose S, Cassaday R, Olomu O, Mendoza A, Yeung C, Gorlick R, Hewitt SM, Helman LJ (2004). The membrane-cytoskeleton linker ezrin is necessary for osteosarcoma metastasis. Nat Med.

[B15] Fievet BT, Gautreau A, Roy C, Del Maestro L, Mangeat P, Louvard D, Arpin M (2004). Phosphoinositide binding and phosphorylation act sequentially in the activation mechanism of ezrin. J Cell Biol.

[B16] Turunen O, Sainio M, Jaaskelainen J, Carpen O, Vaheri A (1998). Structure–function relationships in the ezrin family and the effect of tumor-associated point mutations in neurofibromatosis 2 protein. Biochim Biophys Acta.

[B17] Krieg J, Hunter T (1992). Identification of the two major epidermal growth factor-induced tyrosine phosphorylation sites in the microvillar core protein ezrin. J Biol Chem.

[B18] Elliott BE, Tam SP, Dexter D, Chen ZQ (1992). Capacity of adipose tissue to promote growth and metastasis of a murine mammary carcinoma: effect of estrogen and progesterone. Int J Cancer.

[B19] Elliott BE, Qiao H, Louvard D, Arpin M (2004). Co-operative effect of c-Src and ezrin in deregulation of cell-cell contacts and scattering of mammary carcinoma cells. J Cell Biochem.

[B20] Pantel K, Brakenhoff RH (2004). Dissecting the metastatic cascade. Nat Rev Cancer.

[B21] Ridley AJ, Schwartz MA, Burridge K, Firtel RA, Ginsberg MH, Borisy G, Parsons JT, Horwitz AR (2003). Cell migration: integrating signals from front to back. Science.

[B22] Qiao H, Saulnier R, Patrzykat A, Rahimi N, Raptis L, Rossiter JP, Tremblay E, Elliott B (2000). Cooperative effect of hepatocyte growth factor and fibronectin in anchorage-independent survival of mammary carcinoma cells: requirement for phosphatidylinositol 3-kinase activity. Cell Growth Differ.

[B23] Lin EH, Hui AY, Meens JA, Tremblay EA, Schaefer E, Elliott BE (2004). Disruption of Ca^2+^-dependent cell-matrix adhesion enhances c-Src kinase activity, but causes dissociation of the c-Src/FAK complex and dephosphorylation of tyrosine-577 of FAK in carcinoma cells. Exp Cell Res.

[B24] Srivastava J, Elliott BE, Louvard D, Arpin M (2005). Src-dependent ezrin phosphorylation in adhesion-mediated signaling. Mol Biol Cell.

[B25] Elliott BE, Ekblom P, Pross H, Niemann A, Rubin K (1994). Anti-b1 integrin IgG inhibits pulmonary macrometastasis and the size of micrometastases from a murine mammary carcinoma. Cell Adhes Commun.

[B26] Shaw LM, Chao C, Wewer UM, Mercurio AM (1996). Function of the integrin α6β1 in metastatic breast carcinoma cells assessed by expression of a dominant negative receptor. Cancer Res.

[B27] Dai JL, Wang L, Sahin AA, Broemeling LD, Schutte M, Pan Y (2004). NHERF (Na^+^/H^+ ^exchanger regulatory factor) gene mutations in human breast cancer. Oncogene.

[B28] Voltz JW, Weinman EJ, Shenolikar S (2001). Expanding the role of NHERF, a PDZ-domain containing protein adapter, to growth regulation. Oncogene.

